# Nitrogen Headspace Improves the Extra Virgin Olive Oil Shelf-Life, Preserving Its Functional Properties

**DOI:** 10.3390/antiox8090331

**Published:** 2019-08-22

**Authors:** Antonella Smeriglio, Giovanni Toscano, Marcella Denaro, Clara De Francesco, Simona Agozzino, Domenico Trombetta

**Affiliations:** 1 Department of Chemical, Biological, Pharmaceutical and Environmental Sciences, University of Messina, Viale Palatucci, 98168 Messina, Italy; 2 Foundation Prof. Antonio Imbesi, University of Messina, P.zza Pugliatti 1, 98122 Messina, Italy

**Keywords:** Extra virgin olive oil, Shelf-life, Polyphenols, α-Tocopherol, Fatty acids, Acidity, Peroxides, Dienes, Trienes, Antioxidant power

## Abstract

The functional foods field has recently evolved due to new research being carried out in the food area and greater regulations; these factors have contributed to the creation of health claims, and to the increasing attention that consumers give to health-promoting food products. The aim of this research was to improve the shelf-life of a typical functional food of the Mediterranean diet, the Extra Virgin Olive Oil (EVOO). We focused our attention on the standardization and validation of a production process, starting from the cultivation and harvesting of the olives, which would guarantee a product of quality in terms of bioactive compound content. Furthermore, a methodology/procedure to preserve them in the best way over a long period of time, in order to guarantee the consumer receives a product that retains its functional and organoleptic native properties, was evaluated. The monitoring of biological cultivations, harvesting, milling process, and storage, as well as careful quality control of the analytical parameters (e.g., contents of polyphenols, α-tocopherol, fatty acids, acidity, peroxides, dienes, trienes, ΔK, and antioxidant power) showed that, under the same conditions, a nitrogen headspace is a discriminating factor for the maintenance of the functional properties of EVOO.

## 1. Introduction

Extra virgin olive oil (EVOO) is a functional food obtained by the cold pressing of high-quality *Olea europea* L. fruits produced only during a short period of the year, because the fruit easily loses its qualities over time [1]. 

Cold pressing preserves both the nutritional value of EVOO as well as the natural antioxidants produced in response to environmental stress. Indeed, compared to other vegetable oils, it is not obtained from seeds, dormant organisms with low metabolic activity, but from the fruit, which possess high metabolic activity, conferring greater phytochemical complexity upon the derived product.

EVOO contains more than 98–99% saponifiable fraction (mainly triglycerides) and 1–2% of unsaponifiable fraction, which consist mainly of sterols, squalene, α-tocopherol, and minor compounds such as polyphenols [2], which give it not only organoleptic characteristics, but also different health properties [3,4,5,6,7,8,9].

The quality of the EVOO depends on many factors, such as production process (fruit features, crushing, and malaxation technical variables), as well as storage conditions. It begins with the ripening of the olive and finishes with the packaging. The latter is a very important aspect, because several factors, such as the oxygen availability, as well as temperature and light during storage, can affect the EVOO shelf-life [10]. 

Oxidation is the key factor involved in EVOO quality deterioration. From this point of view, polyphenols, and in particular secoiridoids, together with α-tocopherol, play a pivotal role. The oxidation processes, which can occur during EVOO storage, are photooxidation, thermoxidation, and autoxidation [1]. The first takes place after exposure to light. For example, chlorophylls, which are natural photosensitizers, react with triplet oxygen, a much more reactive species, to form excited state singlet oxygen and, as consequence, the storage and packing conditions of EVOO become of primary importance [11,12]. Thermoxidation occurs when EVOO is exposed to high temperatures [1], while autoxidation occurs when organic compounds react with the available oxygen [13]. This last free radical mechanism-based process is one of the best-known causes of the EVOO alterations during storage [14].

The main susceptible fraction is the lipid one, because its degradation leads to carbonyl and aldehyde compounds conferring off-flavors and oxidative rancidity to the EVOO. In fact, to maintain the designation of EVOO, the lipid oxidation products, such as peroxides or conjugated dienes and trienes, must not exceed maximum threshold limits [15]. 

EVOO stability is also affected by the presence of suspended solids and vegetation water, which cause fermentation and off-flavors with fusty-muddy sediments or winey [16,17]. Filtration or clarification are the main methods used to minimize these events. The European Community (EEC Reg. 1638/1998) approves the first as pre-treatment before bottling [18,19]. It reduces the phospholipid and water content, counteracting the EVOO’s cloudiness during storage and enhancing its stability [18,19]. The clarification technique is based on the insufflation of an inert gas flow from the bottom of the filter tank containing the EVOO directly to the center of the olive oil mass, generating circular bubble movements that enhance the separation of suspended solid particles and vegetation water [20]. 

EVOO quality may also be directly influenced by the primary storage containers, as well as by the final packaging [10]. Indeed, contact with inadequate materials contribute oxidative degradation reactions [21]. For this purpose, the most commonly used containers are tin, steel, or aluminum alloy ones. Furthermore, resins-coated tins are often used to avoid metal surface corrosion [15]. The best final packaging for EVOO is glass; in particular, greenish glass with low UV-light transmittance, which represents a good barrier against light, moisture, and gases [22].

Shelf life is the period from production to consumption, during which the total quality of the product must be maintained. Because fats decompose into different oxidation by-products over time, there is increasing interest in determining EVOO shelf life by studying the occurrence of oxidation processes. EVOO shelf-life has been assessed to be 12–18 months [23], even if it has been shown that when it is properly stored in well-sealed packages, EVOO can reach sustain a second year of storage maintaining its sensorial properties unaltered [24]. Despite the fact that several studies on the accelerated shelf-life of EVOO have been carried out [1,25,26], only few studies are currently available about room-temperature shelf-life, and no data are available about new packaging strategies to preserve the functional properties over time in the best possible manner.

In light of this, the aim of this work was to improve the shelf-life of a biological Sicilian EVOO, produced by a standardized and validated process, which guarantees a product of quality in terms of bioactive compound content, thanks to a new packaging strategy. To this end, a comparison was carried out between EVOO bottled with and without a nitrogen headspace during room temperature long-term storage (18 months), monitoring several parameters such as polyphenols, α-tocopherol, acidity, peroxides, conjugated dienes and trienes, ΔK and antioxidant power.

## 2. Materials and Methods

### 2.1. EVOO Sample Features

Early harvest monovarietal (Tonda Iblea cultivar) organic [27] EVOO, kindly provided by P&P Farma S.r.l. (Turin, Italy), was produced through deep control of the operating parameters of cultivation, harvesting, and cold-pressing in Val Tellaro area (Monti Iblei, Noto–Rosolini–Pachino–Ispica, Sicily, Italy). Automatic irrigation was used for monitoring the cultivation phase. Harvesting by combing was carried out at the end of September–early October, since olives have the highest concentration of antioxidant and aromatic compounds at that time.

After that, in the afternoon hours of the same day of harvesting, the operations of washing, leaf removal, grinding, and cold pressing was carried out. The bulk EVOO was transferred into a stainless steel tank under nitrogen headspace to decant, and then poured, thereby removing all solid residuals. 

Finally, EVOO was poured into amber glass bottles with or without a nitrogen headspace, sealed with a pilfer-proof cap, labelled, packed in a box for storage at room temperature, and immediately shipped to the laboratory, in which it was stored in the dark and away from heat sources until analysis at time at 0, 3, 6, 9, 12, 15 and 18 months (T_0_–T_18_). The modified atmosphere packaging was performed by a station placed downstream from the filling one which dispenses nitrogen gas.

### 2.2. Maturation Index

The olives maturation index (MI) was established depending on the skin and flesh color, from deep green (0) to black (7), according to the method described by Trombetta et al. [28]. The MI was calculated as follows:

MI = a × 0 + b × 1 + c × 2 + d × 3 + e × 4 + f × 5 + g × 6 + h × 7/100(1)

where a, b, c, d, e, f, g, and h represent the fruit numbers of each category. The analysis was repeated three times.

### 2.3. Chemicals

All solvents used were of high purity grade and were purchased from VWR (Milan, Italy). HPLC grade solvents were purchased from Merck (Darmstadt, Germany). Commercial standards, all of proper purity grades, were acquired from Sigma–Aldrich (Milan, Italy) and Extrasynthese (Genay, France). Other chemicals were of analytical grade and were purchased from Sigma–Aldrich (Milan, Italy).

### 2.4. Quality and Oxidation Indices

Free acidity and peroxide value (PV), as well as conjugated dienes and trienes (K_232_ and K_270_, respectively), and ΔK were estimated according to the analytical methods described by the European Community Regulation (EEC) 2568/91 and subsequent amendments [29].

### 2.5. α-Tocopherol Determination

EVOO samples were treated and analyzed following the UNI EN ISO 9936:2011 Official Method [30]. Briefly, 100 mg of each sample was added with 10 mL of n-heptane, filtered through a 0.22 µm nylon syringe filter, and injected into Agilent HPLC system (1100 series, Santa Clara, CA, USA), equipped with a fluorescence detector (FLD) (G1321). Chromatographic elution was performed by LiChrosorb SI-60 (250 × 4.6 mm, 5 µm) column maintained at 25 °C. n-heptane/tetrahydrofurane mixture (96.15/3.85 *v*/*v*), with a flow rate of 1.0 mL/min, was used as mobile phase. Fluorescence detection (λ_ex_ 295 nm, λ_em_ 330 nm) was used to identify and quantify the α-tocopherol content by using an external standard calibration curve. The results were expressed as mg equivalents of α-tocopherol/kg of EVOO.

### 2.6. Fatty Acid Determination

EVOO fatty acid composition was carried out according to Pojic et al. [31]. Briefly, EVOO samples were extracted with chloroform/methanol (2:1 *v*/*v*) mixture and dried by a gentle stream of nitrogen at room temperature (RT). Transesterification with 14% boron (III) fluoride in methanol was carried out in order to obtain fatty acid methyl esters (FAMEs).

Gas chromatographic (GC) analysis was performed on an Agilent gas chromatograph, Model 7890A, equipped with a flame ionization detector (FID) (Agilent Technologies Santa Clara, CA, USA). Elution was carried out by HP-5MS capillary column (30 mm, 0.25 mm coated with 5% diphenyl- and 95% dimethyl-polysiloxane, 0.25 μm film thickness) using helium as carrier gas (1 mL/min, costant flow). The injection was done in split mode (50:1), with an injected volume of 1 μL. The injector and detector temperatures were set at 250 °C and 280 °C, respectively. The oven temperature was held at 50 °C for 2 min, increased to 250 °C at 4 °C/min, and held at 250 °C for 15 min. 

The percentages of compounds were determined from their peak areas in the GC-FID profiles. Gas chromatography-mass spectrometry (GC-MS) analysis was carried out on the above instrument, coupled with an Agilent 5975C mass detector, with the same column and the same operative conditions used for the GC-FID analysis. Ionization voltage was set to 70 eV, the electron multiplier to 900 V, and the ion source temperature to 230 °C. Mass Spectra data were acquired in the scan mode (*m*/*z* 45–450). Detected compounds were identified based on the following parameters: GC retention index (relative to C7–C40 n-alkanes on the HP-5MS column), matching of mass spectral data with those of the MS library (NIST 08), comparison of MS fragmentation patterns with those reported in literature and co-injection with Supelco 37 component fatty acid methyl ester mix (Sigma−Aldrich).

### 2.7. Sample Preparation and Polyphenols Analysis

The EVOO polyphenol fraction was isolated by liquid-liquid extraction, according to Trombetta et al. [28] and then analyzed by RP-LC-DAD analysis. Twenty milliliters of methanol/water mixture (80:20 *v*/*v*) was added, five times, to 10 g of each EVOO sample, vortex-mixed for three minutes, and then the supernatants collected and concentrated by a rotary evaporator (Buchi R-205, Cornaredo (MI), Italy) at RT until a syrupy consistency was obtained. 

Extracts were then added with 10 mL of acetonitrile and defatted three times by adding 10 mL of hexane. The acetonitrile fraction was brought to dryness with a gentle stream of nitrogen. Before injection, the samples were solubilized in methanol and filtered by a 0.2 µm nylon syringe filter.

The qualitative and quantitative determination of polyphenols was carried out using an Agilent HP1100 system (1100 series, Santa Clara, CA, USA), equipped with a photodiode-array detector (DAD) (G1315). The elution was performed by Ascentis 150 × 4.6 mm, 5 µm column (Phenomenex, Macclesfield, UK) by using solvent (A) H_2_O (0.2% CH_3_COOH, pH 3.1) and (B) CH_3_OH according to Trombetta et al., (2017) method. Results were expressed as mg of tyrosol equivalents/kg of EVOO.

### 2.8. Folin-Ciocalteu Assay

The reducing ability of EVOO polyphenol extracts were estimated by the Folin-Ciocalteu method according to Smeriglio et al. [32]. Gallic acid was used as a reference compound. Results were expressed as mg of gallic acid equivalent (GAE)/100 g of EVOO.

### 2.9. ORAC Assay

Free-radical scavenging activity of EVOO polyphenol extracts against AAPH radical was tested according to Bellocco et al. [33]. Briefly, 20 µL of sample were dissolved in 75 mM phosphate buffer (pH 7.4) and pre-incubated for 15 min at 37 °C with 120 µl of fluorescein solution (117 nM). Fresh daily 40 mM AAPH solution (60 µL) was added to the reaction mix and the fluorescence decay recorded every 30 s for 90 min (λ_ex_ 485 nm; λ_em_ 520 nm) by a plate reader (FLUOstar Omega, BMG LABTECH, Offenburg, Germany). A phosphate buffer, instead of a sample, was used as the negative control, while trolox was used as reference compound (10–75 μM). The results are expressed as μmol of trolox equivalents/100 g of EVOO.

### 2.10. Statistical Analysis

The results were expressed as mean ± standard deviation (SD) of three independent experiments (*n* = 3) and analyzed by one-way analysis of variance (ANOVA). The statistically significant difference among averages at the same storage time (0–18 months) was calculated by non-parametric Kruskal- Wallis test followed by Pairwise Multiple Comparison Procedures (Student-Newman-Keuls Method) using SigmaPlot 12.0 software (Systat Software Inc., San Jose, CA). Results were statistically significant at *p* < 0.05.

## 3. Results and Discussion

The monovarietal EVOO evaluated in this study, Tonda Iblea, represents an autochthonous olive cultivar grown in the Eastern part of Sicily (Italy), in which the peculiar microclimate has a significant impact on cultivar features, conferring upon the final product (EVOO) organoleptic and nutritional characteristics which are much appreciated in Italy and abroad.

The maturation index (MI) of the EVOO under study was 1.35 ± 0.01, which perfectly reflects the values found previously in Italian early-harvest, organic, monovarietal EVOOs (1.32–2.26) [28]. Table 1 shows the results of the baseline parameters (T0) which were taken into account to establish the EVOO quality according to European Economic Community (EEC) regulations [29], as well as other parameters, such as polyphenols and α-tocopherol content, which were chosen to evaluate the potential functional properties of the sample under study according to European Food Safety Agency (EFSA) statements [34,35].

The EVOO under study showed an excellent quality, having parameters that fit perfectly in the media or even above average, in terms of EVOO quality, in addition to an excellent fatty acid profile (Table 1). Furthermore, it possessed high polyphenols and α-tocopherol contents (560.06 mg/kg and 217.10 mg/kg, respectively), which perfectly matches EVOOs’ required antioxidant compounds, and is also in accordance with the MI values and organic cultivation [28,36]. 

These results, in addition to the particular olive variety investigated, are attributable to a deep control of the operating parameters of cultivation, harvesting, and cold pressing. It is well-known that during olive crushing and kneading, the enzymatic activity is intense. Oleuropein, for example, is hydrolyzed to the aglycone, which can be metabolized to the dialdehydic form (oleacein) or can be in equilibrium with elenolic acid ester, which can be hydrolyzed to free hydroxytyrosol. Ligstroside, an oleuropein analogue but derivative of tyrosol, follows the same fate. In light of this, production strategies, as well as the collection and processing times, play a key role in baseline EVOO quality.

However, the aim of this study was to evaluate a new strategy to preserve the baseline features of EVOO over time. 

The results of the variation of the main physico-chemical parameters of EVOOs with and without a nitrogen headspace, monitored at different storage times, are reported in Table 2. 

The results showed a decrease of all parameters over time for both EVOOs with or without a nitrogen headspace. Nevertheless, the EVOO with a nitrogen headspace, during storage time, maintained values until the end of the study period (18 months) that perfectly fit those required for EVOO, according to current European regulations [29]. In contrast, EVOO without a nitrogen headspace showed statistically significant differences in all parameters (*p* ≤ 0.05). In particular, the most affected parameters during the storage time result were ΔK (which was outside the limit already after 15 months) and peroxide values (*p* < 0.001 vs EVOO with a nitrogen headspace), although all parameters, with the exclusion of conjugated dienes (K_232_), exceed, even if only slightly, the maximum limits prescribed by European regulation [29] to be included in the EVOO category “stable up to 18 months” (Table 2).

**Figure 1:**
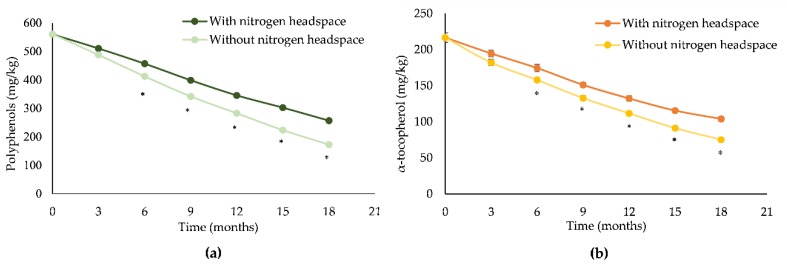
Poyphenols (**a**) and α-tocopherol (**b**) content decay of EVOOs with and without a nitrogen headspace during storage time. *p < 0.001 vs EVOO with a nitrogen headspace.

These results were corroborated by polyphenols and α-tocopherol results, which showed a linear decrease of these antioxidant compounds over time. Furthermore, EVOO without a nitrogen headspace showed a statistically significant decrease (*p* < 0.001) in comparison to the one with a nitrogen headspace that started decreasing from 6 months onwards (Figure 1a,b). 

**Figure 2:**
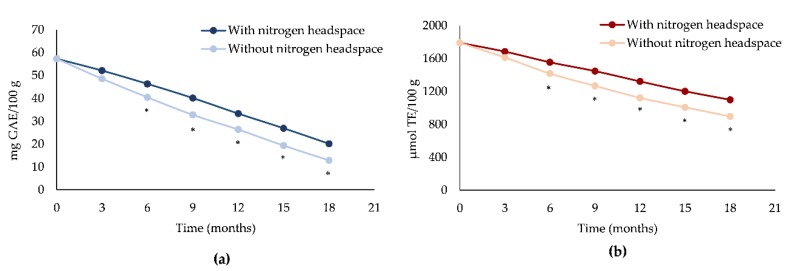
Antioxidant and free-radical scavenging activity decay of EVOOs with or without a nitrogen headspace measured by Folin-Ciocalteu (**a**) and ORAC assay (**b**) during storage time (T_0_–T_18_ months). *p < 0.001 vs EVOO with a nitrogen headspace.

In line with the previous results, also the antioxidant and free-radical scavenging activity of EVOOs decreased substantially over time, with a statistically significant difference (*p* < 0.001) between EVOOs with and without a nitrogen headspace (Figure 2).

This is due in particular to the consumption of bioactive compounds, mainly secoiridoids and α-tocopherol, which possess strong antioxidant properties, in particular thereby reducing the capacity to counteract the oxidative phenomena occurring during the EVOO storage. In contrast, both EVOOs highlighted only a slight decrease in free-radical scavenging activity, as can be observed from Figure 2b, probably due to the hydrolysis of secoiridoids into simpler phenols such as tyrosol and hydroxytyrosol. However, it seems that only the latter is responsible of the maintenance of free-radical scavenging activity due to the presence of a catechol structure with respect to the tyrosol characterized by a mono-phenol structure [37].

Regarding polyphenols in particular, it is interesting to monitor secoiridoid behavior over time. It is well known that secoiridoids are the polyphenols which are mainly implicated in the oxidative preservation of EVOO during storage [38,39,40]. Indeed, as can be observed from Table 3, substantial changes occurred in terms of the relative abundance of secoiridoids over time. 

The so called “oleuropein complex” (hydroxytyrosol derivatives), of which also EFSA talks in its Scientific Opinion [34], as well as tyrosol-derivatives, decrease over time, substantially modifying the polyphenols profile of EVOOs. The most complex secoiridoids are converted during storage into the simplest polyphenols such as tyrosol and hydroxytyrosol, according to previous results [26,39,40], in which a pseudo first-order kinetics equation degradation of secoiridoids during storage independently by temperatures and EVOO varieties was highlighted [26]. In particular, hydroxytyrosol grows exponentially during storage according to previous results, in which it was highlighted that the stability of tyrosol derivatives was greater than that of hydroxytyrosol derivatives [26]. Also in this case, a statistically significant difference (*p* < 0.001) between EVOOs with and without a nitrogen headspace was observed (Table 3).

## 4. Conclusions

This is the first study to investigate a new packaging strategy, the nitrogen bottle headspace, in order to preserve the shelf-life of a biological Sicilian EVOO produced by a standardized and validated process, in order to guarantee a product of quality in terms of bioactive compound content. Furthermore, this is the first time that a room-temperature shelf-life of 18 months, examining all the parameters required by the current legislation for the classification of the product as extra virgin olive oil, as well as the content of α-tocopherol and polyphenols, were tested on EVOOs with and without a nitrogen headspace.

In light of the results, hydroxytyrosol, tyrosol, and secoiridoids contribute significantly to the shelf-life of EVOO more than α-tocopherol, whose degradation occurs much more rapidly. Despite the decrease of secoiridoids over time, polyphenol content remains high, due to the formation of degradation products such as tyrosol and hydroxytyrosol from more complex molecules. A linear relationship exists between the secoiridoids and α-tocopherol content and the oxidative stability of EVOO in terms of resistance to autoxidation, both with and without oxygen availability (nitrogen headspace). 

The monitoring of the cultivation process allowed us to critically evaluate the nutritional and non-nutritional values found, identifying the particular features that will enable the achievement of an EVOO with functional and organoleptic characteristics which is suitable for the conferment of the health claim. 

However, it is not sufficient for the EVOO to possess the features that define it as functional food in advance, because a true functional food is one that possesses these features when consumed.

In order to obtain this, it is necessary to control and standardize all processes to which the food is subjected along the entire production and storage chain.

Finally, this work demonstrates that the insufflation of an inert gas such as nitrogen in the bottle headspace can surely increase the EVOO shelf-life, preserving its organoleptic and functional features throughout the marketing life, which generally varies between 18 and 24 months.

## Figures and Tables

**Table 1:** Baseline parameters (T_0_) of extra virgin olive oil before bottling with or without a nitrogen headspace.

Parameter	Value	SD ^1^	Limit ^2^
Free fatty acids (Oleic acid %)	0.22	0.012	0.8
Peroxyde number (mEq. O_2_/kg of EVOO)	7.75	0.240	20
Conjugated dienes (K_232_)	1.60	0.032	2.5
Conjugated trienes (K_270_)	0.12	0.005	0.22
ΔK	0.001	0.000	0.01
Fatty acid content (%)			
C10:0–Capric acid	≤LOD ^3^	-	-
C12:0–Lauric acid	≤LOD ^3^	-	-
C14:0–Myristic acid	≤LOD ^3^	-	0.03
C16:0–Palmitic acid	10.44	0.237	7.5–20
C16:1n7–Palmitoleic acid	0.85	0.042	0.3–3.5
C17:0–Heptadecanoic acid	0.11	0.005	0.4
C17:1–Heptadecenoic acid	0,15	0.006	0.6
C18:0–Stearic acid	0.65	0.032	0.5–5
C18:1n9–Oleic acid	67.42	2.220	55–83
C18:1n7–Vaccenic acid	≤LOD ^3^	-	-
C18:2n6–Linoleic acid	14.80	0.641	2.5–21
C18:3n3–Linolenic acid	0.55	0.011	1.0
C20:0–Arachidic acid	0.25	0.018	0.6
C20:1n9–Eicosenoic acid	0.22	0.016	0.5
C22:0–Behenic acid	0.12	0.008	0.2
C22:1–Erucic acid	≤LOD ^3^	-	-
C24:0–Lignoceric acid	0.11	0.007	0.2
C24:1n9–Nervonic acid	0.78	0.054	-
Trans fatty acid isomers (%)			
C18:1–(Elaidinic acid)	≤LOD ^3^	-	0.05
C18:2 + C18:3	≤LOD ^3^	-	0.05
Polyphenols (mg of tyrosol/kg of EVOO)	560.06	-	-
Hydroxytyrosol	1.27	0.010	-
Tyrosol	1.25	0.008	-
3,4-DHPEA-AC ^4^	1.84	0.105	-
3,4-DHPEA-EDA ^5^	95.34	2.246	-
p-HPEA-EDA ^6^	22.94	0.552	-
Oleuropein	0.33	0.015	-
3,4-DHPEA-EA ^7^	8.99	0.442	-
α-Tocopherol (mg/kg of EVOO)	217.10	6.554	-

^1^ SD, standard deviation; ^2^ Limit reported refer to EEC regulation [29]; ^3^ LOD, limit of detection; ^4^ 3,4-DHPEA-AC, hydroxytyrosol acetate; ^5^ 3,4-DHPEA-EDA, oleacein; ^6^ p-HPEA-EDA, oleocanthal; ^7^ 3,4- DHPEA-EA, oleuropein aglycone.

**Table 2:** Variations of the main physico-chemical properties of EVOOs with and without a nitrogen headspace at different storage times (months). Data are expressed as mean value ± SD (*n* = 5).

**EVOO with a Nitrogen Headspace**					
**Time**	**FFA ^1^**	**K_232_**	**K_270_**	**ΔK**	**PV^2^**
0	0.22 ± 0.01	1.601 ± 0.01	0.116 ± 0.001	0.001 ± 0.000	7.72 ± 0.2
3	0.29 ± 0.02	1.703 ± 0.01	0.128 ± 0.002	0.002 ± 0.001	8.46 ± 0.1
6	0.37 ± 0.01	1.753 ± 0.03	0.143 ± 0.001	0.002 ± 0.001	10.02 ± 0.3
9	0.42 ± 0.00	1.834 ± 0.01	0.156 ± 0.002	0.003 ± 0.000	12.14 ± 0.2
12	0.48 ± 0.01	1.971 ± 0.01	0.167 ± 0.001	0.004 ± 0.002	13.96 ± 0.l
15	0.56 ± 0.02	2.213 ± 0.01	0.175 ± 0.002	0.005 ± 0.001	15.28 ± 0.2
18	0.61 ± 0.01	2.342 ± 0.01	0.189 ± 0.000	0.006 ± 0.002	17.16 ± 0.2
**EVOO without a Nitrogen Headspace**					
**Time**	**FFA ^1^**	**K_232_**	**K_270_**	**ΔK**	**PV^2^**
0	0.22 ± 0.01	1.601 ± 0.01	0.116 ± 0.001	0.001 ±0.000	7.72 ± 0.2
3	0.34 ± 0.01	1.725 ± 0.02	0.134 ± 0.002	0.002 ± 0.002	9.55 ± 0.1
6	0.42 ± 0.03	1.816 ± 0.00	0.150 ± 0.001	0.004 ± 0.001 *	11.39 ± 0.2 **
9	0.52 ± 0.02	1.892 ± 0.01	0.165 ± 0.003 *	0.008 ± 0.000 **	14.55 ± 0.3 **
12	0.64 ± 0.01 *	2.154 ± 0.02 *	0.188 ± 0.000 *	0.010 ± 0.001 **	16.66 ± 0.2 **
15	0.75 ± 0.01 *	2.355 ± 0.01 *	0.198 ± 0.001 *	0.014 ± 0.000 **	19.12 ± 0.3 **
18	0.85 ± 0.02 *	2.477 ± 0.00 *	0.245 ± 0.001 *	0.018 ± 0.002 **	22.12 ± 0.2 **

FFA = Free Fatty Acid expressed as oleic acid %; ^2^ PV = Peroxide value expressed as mEq. O_2_/kg EVOO; *p < 0.05 vs EVOO with a nitrogen headspace; ** *p* < 0.001 vs EVOO with a nitrogen headspace.

**Table 3:** Hydroxytyrosol, tyrosol and secoiridoids modifications (mg kg^−1^) during storage time (0–18 months) of EVOOs with and without a nitrogen headspace.

**EVOOs with a Nitrogen Headspace**							
**Time**	**Ht ^1^**	**Ty ^2^**	**3,4-DHPEA-AC ^3^**	**3,4-DHPEA-EDA ^4^**	**p-HPEA-EDA ^5^**	**Oleuropein**	**3,4-DHPEA-EA ^6^**
0	1.25 ± 0.05	1.23 ± 0.01	1.86 ± 0.03	95.70 ± 0.80	23.28 ± 0.25	0.34 ± 0.01	9.23 ± 0.35
3	2.10 ± 0.05	2.34 ± 0.00	1.75 ± 0.01	81.95 ± 1.43	20.05 ± 0.97	0.31 ± 0.01	8.08 ± 0.09
6	5.13 ± 0.04	5.13 ± 0.06	1.65 ± 0.01	65.51 ± 0.92	17.02 ± 0.16	0.27 ± 0.04	7.20 ± 0.05
9	15.67 ± 0.34	11.97 ± 0.36	1.54 ± 0.01	49.74 ± 1.17	13.73 ± 0.13	0.22 ± 0.02	6.12 ± 0.06
12	28.01 ± 0.82	22.44 ± 1.10	1.45 ± 0.00	36.06 ± 0.57	10.52 ± 0.36	0.18 ± 0.01	5.11 ± 0.05
15	37.35 ± 1.03	36.35 ± 0.95	1.32 ± 0.02	23.67 ± 0.81	7.65 ± 0.25	0.14 ± 0.00	3.98 ± 0.16
18	46.06 ± 0.59	54.60 ± 1.32	1.19 ± 0.00	14.09 ± 1.03	5.08 ± 0.54	0.12 ± 0.01	3.05 ± 0.11
**EVOOs without a Nitrogen Headspace**							
**Time**	**Ht ^1^**	**Ty ^2^**	**3,4-DHPEA-AC ^3^**	**3,4-DHPEA-EDA ^4^**	**p-HPEA-EDA ^5^**	**Oleuropein**	**3,4-DHPEA-EA ^6^**
0	1.25 ± 0.05	1.23 ± 0.01	1.86 ± 0.03	95.70 ± 0.80	23.28 ± 0.25	0.34 ± 0.01	9.23 ± 0.35
3	2.28 ± 0.01	2.65 ± 0.03	1.71 ± 0.05	78.60 ± 0.53 *	19.52 ± 0.41	0.30 ± 0.02	7.89 ±0.06
6	7.42 ± 0.15 *	7.52 ± 0.05 *	1.60 ± 0.02 *	60.05 ± 1.52 **	15.72 ± 0.16 **	0.24 ± 0.03	6.77 ± 0.29
9	19.19 ± 0.11 *	17.17 ± 0.88 *	1.48 ± 0.03 *	41.43 ± 1.19 **	12.00 ± 0.16 **	0.20 ± 0.02 *	5.43 ± 0.10 *
12	36.79 ± 2.25 *	29.27 ± 0.22 *	1.37 ± 0.01 *	26.59 ± 1.28 **	8.57 ± 0.25 **	0.15 ± 0.01 *	4.17 ± 0.06 *
15	48.82 ± 0.47 *	45.95 ± 2.74 *	1.24 ± 0.01 *	14.03 ± 0.25 **	5.92 ± 0.19 **	0.11 ± 0.01 *	2.91 ± 0.04 *
18	59.41 ± 1.40 *	69.16 ± 0.35 *	1.13 ± 0.00 *	4.17 ± 0.42 **	3.45 ± 0.37 **	0.07 ± 0.00 *	1.87 ± 0.09 *

^1^ Hydroxytyrosol; ^2^ Tyrosol; ^3^ Hydroxytyrosol acetate; ^4^ Oleacein; ^5^ Oleocanthal; ^6^ Oleuropein aglycon. *p < 0.05 vs EVOO with a nitrogen headspace; ** *p* < 0.001 vs EVOO with a nitrogen headspace.
